# The Effect of the Pd Precursors on the Shape of Hollow Ag–Pd Alloy Nanoparticles Using Ag Nanocubes as Seeds

**DOI:** 10.1021/acs.langmuir.3c00799

**Published:** 2023-07-28

**Authors:** Xin Wen, Seyed Amirabbas Nazemi, Robson Rosa da Silva, Kasper Moth-Poulsen

**Affiliations:** † Department of Chemistry and Chemical Engineering, Chalmers University of Technology, SE-412-96 Gothenburg, Sweden; ‡ Department of Physics, Engineering, Earth, Environmental sciences, and Mechanics, University of Grenoble Alpes, 38400 Saint Martin d’Hères, France; § School of Life Science, University of Applied Sciences and Arts Northwestern Switzerlanz, Hofackerstrasee 30, Muttenz CH-4132, Switzerland; ∥ NanoScientifica Scandinavia AB, Stena Center, Studio 4166, 41 292 Gothenburg, Sweden; ⊥ The Institute of Materials Science of Barcelona, ICMAB-CSIC, Bellaterra, 08193 Barcelona, Spain; # Catalan Institution for Research & Advanced Studies, ICREA, Pg. Llu′ıs Companys 23, 08010 Barcelona, Spain; ∇ Department of Chemical Engineering, Universitat Politècnica de Catalunya, EEBE, Eduard Maristany 10−14, 08019 Barcelona, Spain

## Abstract

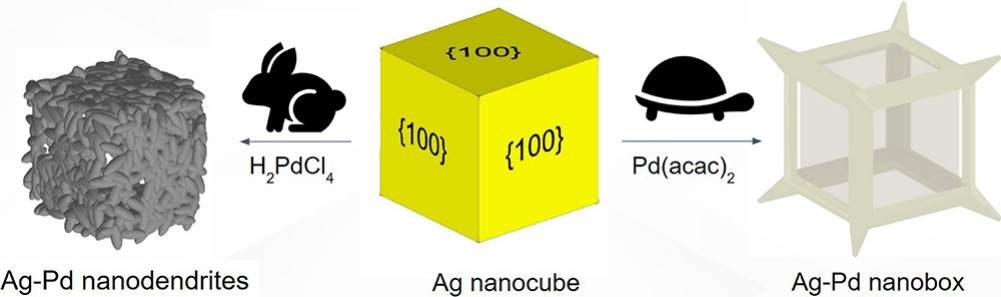

Hollow Ag–Pd nanoparticles have potentially high catalytic performance owing to their larger surface area compared to their corresponding solid nanoparticles. We successfully fabricated hollow Ag–Pd alloy nanodendrites and nanoboxes by using different Pd precursors (H_2_PdCl_4_ and Pd(acac)_2_) to achieve large surface area nanoboxes. Interestingly, the use of a H_2_PdCl_4_ precursor led to the formation of hollow nanodendrite structures, whereas the slower reduction of Pd(acac)_2_ led to the formation of hollow nanoboxes. The microstructure and chemical composition of Ag–Pd nanoparticles and properties of their growth solutions were investigated by transmission electron microscopy, energy-dispersive X-ray spectroscopy, and ultraviolet–visible spectroscopy.

## Introduction

Hollow nanostructures have been studied in different metallic materials. As compared to their solid counterparts, hollow nanoparticles have lower weight and higher surface area, which potentially improves the mass efficiency of noble metallic materials and their properties.^1−3^ Catalytic activity is one of the most important properties of metallic nanoparticles.^4,5^ The interior empty space of hollow nanoparticles enlarges the active surface area, making the nanoparticles highly accessible for reactants.^6,7^ Particularly, the interior catalytic sites are not impressionable to aggregation of the nanoparticles, which could enhance the catalytic activity further.^3^ Additionally, in hollow catalysts, the reactants often access the inner surface via diffusion through the shell of hollow nanoparticles. The diffusion rate depends on the porosity and thickness of the hollow shell and properties of reactants. Therefore, controlling the hollow structure can improve selectivity of catalytic reactions.^8^ Surface plasmon resonance, another important property of noble metallic nanoparticles, can be influenced by the existence of hollow structures in the nanoparticles, which is of high importance for their applications in photonic devices and drug delivery.^2,9^

Studies on hollow palladium (Pd) nanoparticles attract considerable attention as Pd is one of the most valuable metals and its nanoparticles have high catalytic abilities on carbon–carbon coupling and hydrogenation reactions.^10,11^ Carbon–carbon coupling reactions are often used in the synthesis of pharmaceutical compounds in industry, which has led to an increased utilization of Pd as a catalyst in last 20 years.^12^ The high hydrogen-adsorption ability of Pd makes it an excellent candidate as a sensing material for hydrogen gas sensor and hydrogen storage.^13,14^ Nowadays, new catalytic technologies prioritize green catalysts, preventing from human impact at the beginning, rather than environmental remediation after contamination. The catalytic properties could be improved by tailoring the electronic structure of catalytic materials through modification of their microstructure and composition.^15^ Therefore, the synthesis process and structure of materials are important for realization of green catalysts.

One of the most common synthesis methods for hollow Ag–Pd nanoparticles is the double-template method where a sacrificial nanomaterial is overgrown with Pd and oxidized in the process, through galvanic replacement. This method is advantageous in a number of aspects: (a) the synthesis process is simple, versatile, and environmentally friendly; (b) reaction conditions are mild; (c) the size and shape of Ag–Pd nanoparticles are easily controlled; and (d) the synthesis process is highly reproducible.^16,17^ By this approach, a type of metal nanoparticle is used as the hard template and sacrificed through a galvanic replacement reaction with Pd ions from different Pd precursors.^18^ It requires the template metal to have a lower redox potential in order to undergo spontaneous redox reaction between surficial metal template atoms and Pd ions.^19^ For instance, the template could be nickel (Ni) for hollow Pd–Ni alloy nanoparticles,^2,20^ copper(I) oxide (Cu_2_O) for Ag–Pd nanospheres,^21^ and silver (Ag) for Ag–Pd alloy nanoparticles. As Ag is comparatively cheap and easy to get oxidized under reaction conditions, it was selected as a sacrificial template in our experiments. In the double-template method, surfactants or polymers can be used as a soft template to stabilize the shape and size of hollow metallic nanoparticles.^18^ Therefore, the shape can be influenced by using different surfactants. For example, hexadecyltrimethylammonium bromide (CTAB) is often used to synthesize Ag–Pd nanocubes, while Ag–Pd nanodendrites can be obtained by using a mixture of hexadecyltrimethylammonium chloride (CTAC) and sodium oleate (NaOL).^22,23^ Additionally, parameters such as pH value, reaction temperature, and concentrations of reagents can affect the shape and size of Ag–Pd nanoparticles.^24^

Even though some studies have been done on the synthesis and catalytic properties of hollow Ag–Pd nanoparticles, there are no comprehensive studies on the effects of Pd precursors on the formation of hollow Ag–Pd nanoparticles.^25−27^ Since different Pd salts perform a variety of physical and chemical properties, the selection of Pd precursors may influence the synthesis process and the final shape of nanoparticles. Additionally, the catalytic properties vary with crystal facets of hollow Ag–Pd nanoparticles and the facets with high catalytic activity are expected to be formed.^28^ Therefore, it is necessary to study the effects of Pd precursors in shape, physical, and catalytic properties of the hollow Ag–Pd nanoparticles.

In this work, we used dihydrogen tetrachloropalladate (H_2_PdCl_4_) and palladium(II) acetylacetonate (Pd(acac)_2_) as precursors and Ag nanocubes as a hard template to synthesize hollow Ag–Pd nanoparticles with different shapes. The microstructure and chemical composition of these hollow Ag–Pd nanoparticles have been investigated by transmission electron microscopy.

## Experimental Section

### Materials

Palladium(II) chloride (PdCl_2_, 99%), palladium(II) acetylacetonate (Pd(acac)_2_, 99%), silver trifluoroacetate (CF_3_COOAg, 98%), polyvinylpyrrolidone (PVP, *M*_w_ = 55,000), l-ascorbic acid (AA, 99%), sodium hydrosulfide hydrate (NaHS·*x*H_2_O), and hydrochloric acid (HCl, 37 wt % in water) were purchased from Sigma-Aldrich. NaOL (>97.0%) was obtained from Tokyo Chemical Industry. CTAC (99%) was purchased from Acros Organics of Fisher Scientific. Acetone (≥99.8%) and methanol (≥99.9%) were obtained from Fisher Scientific. Ethylene glycol (EG, ≥99%) was purchased from J.T.Baker Chemical (Avantor Performance Materials, LLC).

The Milli-Q water used in the solutions is ultrapure water 18.2MΩ^.^cm purified with a Milli-Q Advantage A10 water purification system from Merck. 10 mM of H_2_PdCl_4_ solution was prepared by dissolving 88.65 mg of PdCl_2_ in 50 mL of 20 mM HCl in a water bath at 60 °C. As Pd(acac)_2_ is insoluble in water, 10 mM of Pd(acac)_2_ solution was freshly prepared by adding 15.23 mg of Pd(acac)_2_ in 5 mL of Milli-Q water and mixing for several seconds on a vortex followed by the addition of 10 mL of methanol. The mixture was mixed thoroughly on the vortex for 5 min and added in the reaction directly to prevent Pd ions from reduction of methanol. Additionally, CTAC and NaOL aqueous solutions were stored in a water bath at 40 °C until use.

### Synthesis of Ag Nanocubes

The synthesis of the Ag nanocubes was initially adapted from the method described by Zhang et al.^29^ and Wand et al.^30^ All reagents were prepared in EG solvent.

Briefly, 50 mL of EG was added into a 250 mL round-bottom flask and incubated in an oil bath at 150 °C for 1 h, while the solution was stirred at a speed of 550 rpm. Then, 0.6 mL of 3 mM NaHS was injected in the heated solution. After 4 min, 5 mL of 3 mM HCl solution was added. 2 min later, 12.5 mL of PVP solution (20 mg/mL) was added in the flask. After another 2 min, 4 mL of 282 mM CF_3_COOAg was injected. The reaction solution was incubated at 150 °C for 30 min, while stirring continuously. During the entire process, the outlet of the flask was sealed except for the time of reagent addition. Finally, the round-bottom flask containing the prepared Ag nanocubes was placed into an ice bath to quench the reaction and stop the further growth of Ag nanocubes.

For purification, 7.5 mL of prepared Ag nanocube suspension was mixed with 42.5 mL of acetone in a 50 mL centrifuge tube and then centrifuged at 4900 rpm (2797 RCF) for 8 min. After removing the supernatant, Ag nanocubes were dispersed in 1.5 mL of Milli-Q water using the vortex and ultrasonication and then transferred into a 1.5 mL Eppendorf centrifuge tube. The Ag nanocube suspension was centrifuged (VWR Micro Star 12) again at 13,500 rpm (12,300 RCF) for 15 min. 1 mL of Milli-Q water was added after the supernatant was removed. This Ag nanocube suspension was stored at room temperature for future use.

### Synthesis of Hollow Ag–Pd Nanodendrites

The growth of Ag–Pd nanodendrites on Ag nanocubes was adapted and modified based on our previously reported method.^23^ An optimal molar ratio of CTAC and NaOL (4:1) was applied in our synthesis. 4 mL of 50 mM CTAC and 1 mL of 50 mM NaOL aqueous solutions were mixed well in a 20 mL glass vial. After that, 25 μL of Ag nanocube suspension was added as seeds. Finally, 25 μL of 100 mM AA and 125 μL of 10 mM H_2_PdCl_4_ solutions were added in sequence. This solution mixture was mixed thoroughly on the vortex and then transferred to a water bath at 40 °C for 4 h without stirring. The final product was centrifuged at 12,000 rpm (9700 RCF) for 10 min. After removing the supernatant, the hollow Ag–Pd nanodendrites were dispersed in the same volume of Milli-Q water.

### Synthesis of Hollow Ag–Pd Spiky Nanoboxes

The synthesis of hollow Ag–Pd spiky nanoboxes was followed by the same procedure of the synthesis of hollow Ag–Pd nanodendrites, but this time, 375 μL of Pd(acac)_2_ in the mixture of water and methanol was used as a Pd source, which contained the same amount of Pd ions as H_2_PdCl_4_ in the growth solution of hollow Ag–Pd nanodendrites. Since the Ag–Pd nanoboxes grew more slowly than the Ag–Pd nanodendrites (Figure S1 of the ESI for more details), the mixed growth solution for hollow Ag–Pd nanoboxes was incubated in a water bath at 40 °C for longer hours (22 h).

### Characterization

UV–visible extinction spectra were taken on an Agilent Cary 100 UV–vis spectrophotometer at room temperature. The zeta potential of nanoparticle suspensions was investigated on a Malvern Panalytical Zetasizer Nano ZS. The microstructure of nanoparticles was studied with a FEI Tecnai T20 transmission electron microscope (TEM) at 200 kV, FEI Titan 80–300 TEM at 300 kV, and a JEOL JEM-1200EX II TEM at 120 kV.

## Results and Discussion

**Figure 1:**
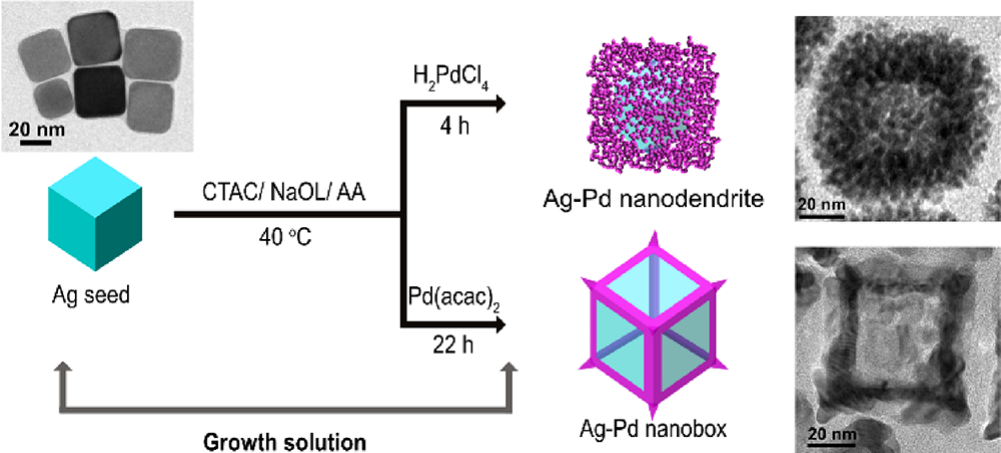
Schematic illustration of the synthesis process of hollow Ag–Ag–Pd nanodendrites and Ag–Ag–Pd nanoboxes. TEM images of Ag nanocubes, hollow Ag–Pd nanodendrites, and hollow Ag–Pd nanoboxes are inserted next to their models.

**Figure 2:**
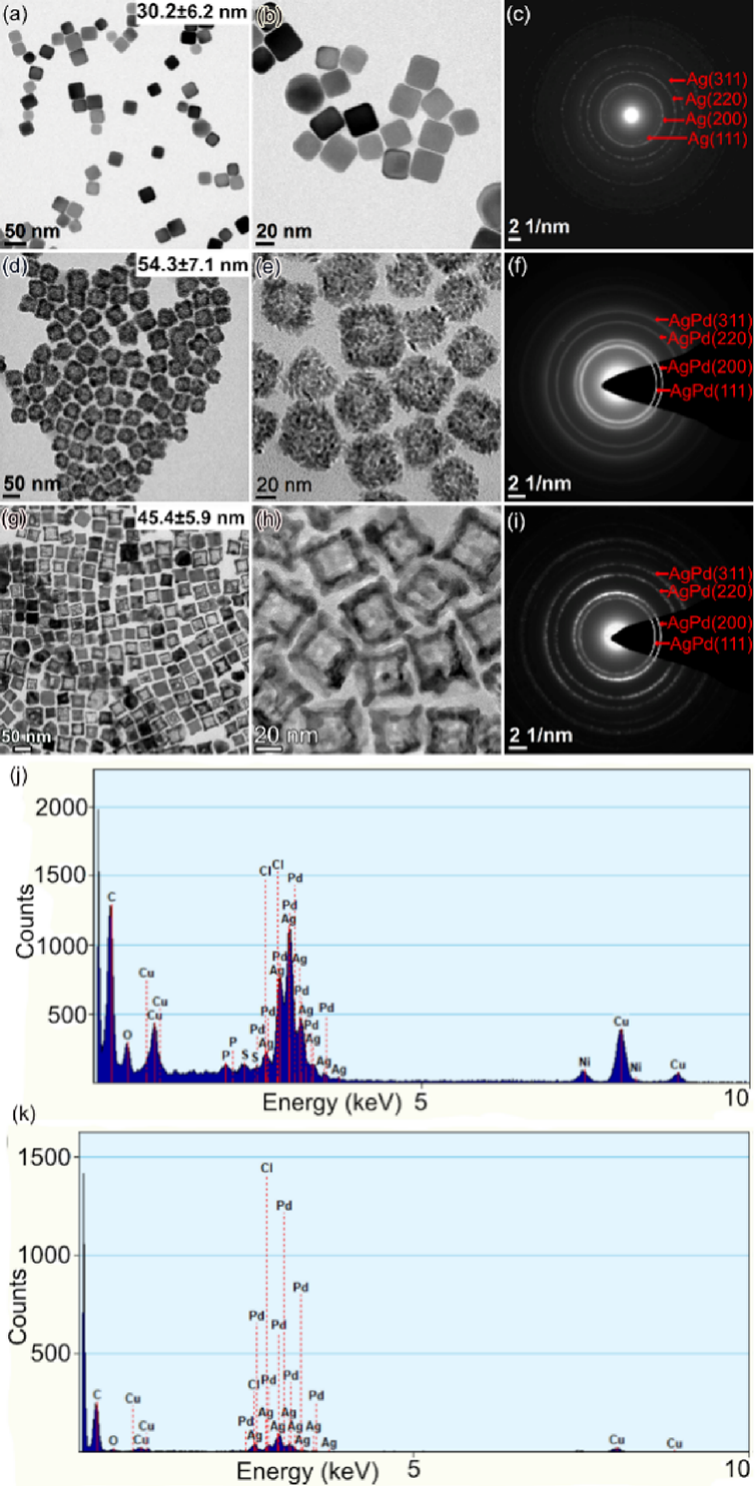
Low-magnification TEM images (LM), high-magnification images (HM), and selected area electron diffraction patterns (SAED) are shown in sequence. (a) LM, (b) HM, and (c) SAED for Ag nanocubes. (d) LM, (e) HM, and (f) SAED for Ag–Pd nanodendrites. (g) LM, (h) HM, and (i) SAED for Ag–Pd nanoboxes. The average size of each nanoparticle is marked in the corresponding low-magnification TEM image. Histograms of the size distribution are shown in Figure S2 of ESI. (j) Energy-dispersive X-ray spectroscopy (EDS) spectrum of Ag–Pd nanodendrites. (k) EDS spectrum of Ag–Pd nanoboxes.

In our experiments, H_2_PdCl_4_ and Pd(acac)_2_ were used as Pd precursors, respectively, in the seed-mediated growth of hollow Ag–Pd nanoparticles, where the Ag seeds were sacrificed by a galvanic replacement reaction with the Pd precursors. The schematic illustration of this process is displayed in Figure 1. A certain amount of Ag nanocubes was added as the seeds in the growth solution containing one type of Pd precursors, CTAC and NaOL as capping agents, and AA as a reducing agent. Where H_2_PdCl_4_ was employed, cubic Ag–Pd nanodendrites were formed after a 4 h growth. An empty cubic core with similar size and shape with the Ag seed can be observed by contrast in the corresponding TEM images (Figure 2). However, where Pd(acac)_2_ was used as the precursor, hollow Ag–Pd nanoboxes were formed. The empty cubic core can be clearly detected by contrast in TEM images (Figure 2). Moreover, four corners of these nanoboxes grew further forming spiky tips.

The quality of these Ag nanocubes, hollow Ag–Pd nanodendrites, and nanoboxes was investigated by TEM imaging at different magnifications and selected area electron diffraction (SAED) patterns, shown in Figure 2. The TEM images of Ag nanocubes in Figure 2a,b indicate that a high yield of cubic Ag seeds with an average size of 30.2 ± 6.2 nm has been produced. The diffraction rings from inner to outer ones in the SAED pattern of these Ag nanocubes correspond to (111), (200), (220), and (311) lattice planes of Ag crystals (marked in Figure 2c), respectively, verifying that these Ag nanocubes consist of Ag crystals.

TEM images of hollow Ag–Pd nanodendrites presented in Figure 2d,e suggest a high yield and uniform shape with a measured average size of 54.3 ± 7.1 nm. The TEM images of hollow Ag–Pd nanoboxes (Figure 2g,h) also verify them to have a high yield and uniform shape. The average size of the Ag–Pd nanoboxes is measured to be 45.4 ± 5.9 nm, slightly smaller than the Ag–Pd nanodendrites. SAED patterns of hollow Ag–Pd nanodendrites and nanoboxes were produced to identify their crystal structures, as shown in Figure 2f,i. SAED patterns of both nanoparticles present classical polycrystal diffraction characteristics of face-centered cubic (FCC) structure. However, the interplanar crystal spacings of (111), (200), (220), and (311) lattice planes measured from the SAED patterns are slightly larger than the values of Pd crystals, which raises our suspicion on the chemical composition of these hollow Ag–Pd nanodendrites and nanoboxes. Therefore, energy-dispersive X-ray spectroscopy (EDS) was performed on these two hollow nanostructures and the results are shown in Figure 2j,k. Strong peaks of Ag and Pd elements are discovered in both EDS spectra, except for the peaks of Cu and C from the TEM grids and the elements from reaction reagents such as Cl and S. This indicates that a portion of Ag remains in the lattice of hollow Pd structures. Additionally, an average atomic percent of Ag in the Ag–Pd nanostructure was calculated by performing EDS on three more regions (shown in Table S1 of the ESI). The results verify that hollow Ag–Pd nanodendrites consist of 43% of Pd and 57% of Ag and hollow Ag–Pd nanoboxes contain 41% of Pd and 59% of Ag.

**Figure 3:**
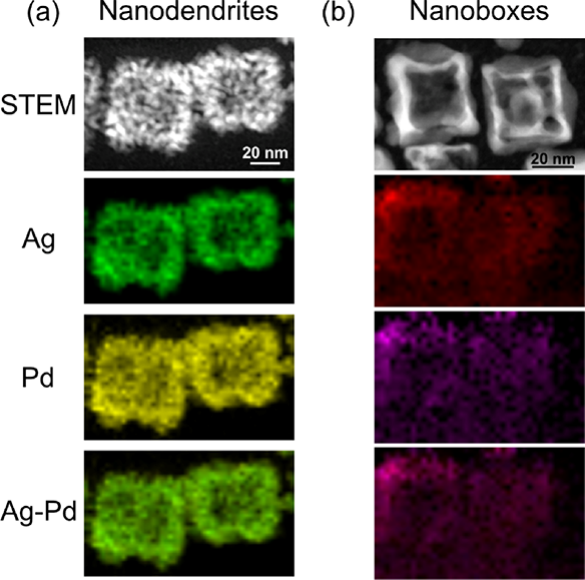
EDS mapping of hollow Ag–Pd nanoparticles, including a scanning transmission electron microscope (STEM) image of the particles and mappings of Ag, Pd, and Ag–Pd overlay. (a) Nanodendrites, where green is Ag and yellow is Pd. (b) Nanoboxes, where red is Ag and purple is Pd.

Even though the EDS spectra in Figure 2 confirm that these hollow Ag–Pd nanoparticles contain a high percent of Ag, it is still unclear if Ag and Pd elements form a core–shell structure or alloy. Therefore, further EDS mapping was performed to investigate the distribution of Ag and Pd and the results are depicted in Figure 3. The mappings of Ag and Pd in Figure 3a indicate that both elements distribute over the entire nanodendrites. The signals of Ag and Pd are mixed evenly, and no core–shell structure can be observed from the mapping of Ag–Pd overlay. However, comparing the signal intensity, signified by the color intensity, between the edge and center regions, both Ag and Pd have stronger signals at the edge than the center of the nanodendrites, indicating the existence of the empty cores, which is consistent with the observation on TEM and STEM images. The same result is obtained from the EDS mapping of hollow Ag–Pd nanoboxes in Figure 3b. Ag and Pd elements are mixed evenly and distributed over the whole nanoboxes. The empty cores in the center of the particles can be observed by the signal intensity difference in the mappings of Ag, Pd, and Ag–Pd overlay. Therefore, the results in Figure 3 verify that the nanoparticles synthesized in this work are hollow Ag–Pd alloys.

**Figure 4:**
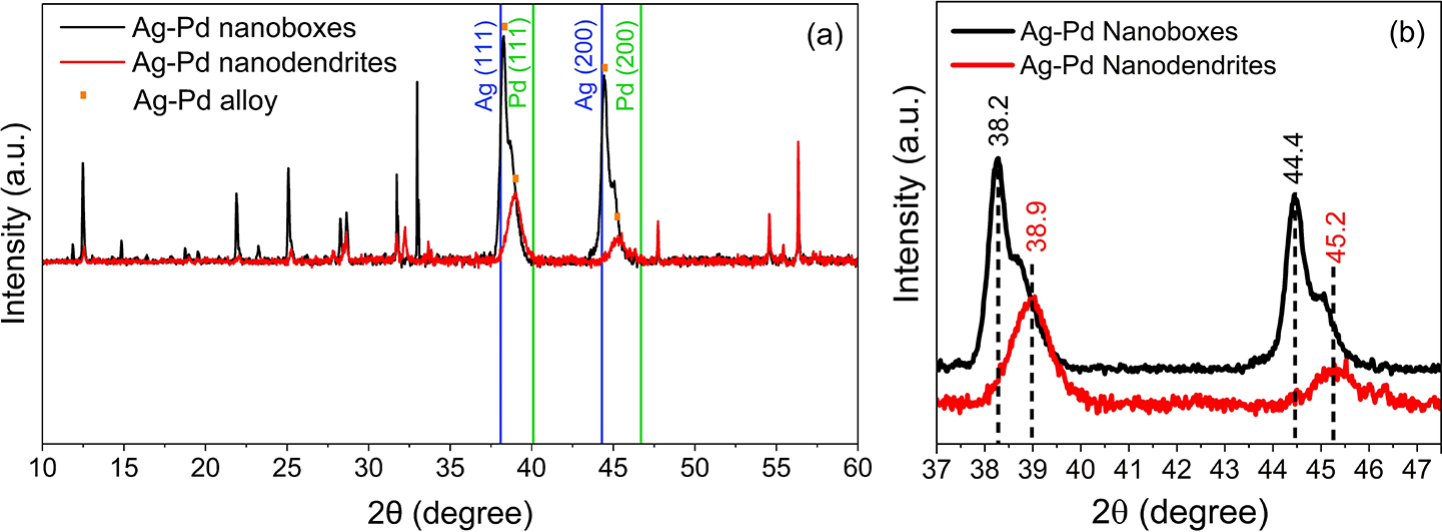
X-ray diffraction pattern of Ag-Pd the Ag−Pd nanoboxes and nanodendrites (a) and, a zoomed view of the spectra in the region 37°−48° (b). Reference peaks are shown in green and blue lines.

In order to further verify the crystalline structure of the nanoparticles, X-ray powder diffraction (XRD) was conducted for both nanoboxes and nanodendrites. The results are presented in Figure 4.

The XRD measurement reveals a homogenous alloy structure of the Ag–Pd nanodendrites with a 50/50 fraction of each, while the spectrum of the nanoboxes indicates that there is a two-fold Ag-rich fraction compared to Pd. As can be seen, there are few peaks present in the measurements, which can be explained by the unreacted chemicals and/or unbound ligands within the samples. This set of results along with EDS measurements verifies the crystalline structure of the Ag–Pd nanoboxes and nanodendrite alloys.

**Figure 5:**
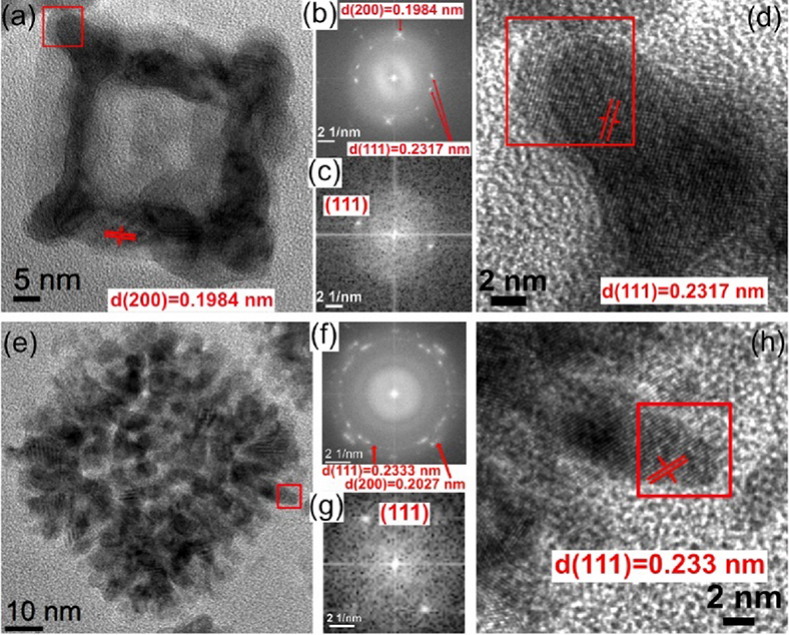
(a) High-resolution TEM image of an entire hollow Ag–Pd nanobox. The (200) lattice plane is noted by a pair of parallel red lines, and its interplanar spacing is recorded in the image. (b) The corresponding FFT of (a). Interplanar spacings of (111) and (200) lattice planes are marked in the image. (c) The FFT of (d). (d) High-resolution TEM image of the spiky corner, marked by a red square in (a). The (111) lattice plane and its interplanar spacing are recorded. (e) High-resolution TEM image of an entire hollow Ag–Pd nanodendrite. (f) The corresponding FFT of (e). The interplanar spacings of (111) and (200) lattice planes are recorded in the image. (g) The FFT of (h). (h) High-resolution TEM image of the dendrite marked by a red square in (e). The (111) lattice plane is marked by a pair of parallel red lines, and its interplanar spacing is recorded in the image.

High-resolution TEM was carried out at different magnifications to investigate the microstructure of hollow Ag–Pd nanodendrites and nanoboxes in detail. Figure 5a displays a high-resolution TEM image of a single Ag–Pd nanobox. The Fast Fourier transform (FFT) image presented in Figure 5b shows a polycrystal characteristic, but the spots of (200) and (111) lattice planes are clear and have strong intensities, illustrating that the Ag–Pd nanobox consists of several crystals. (200) lattice planes are marked by a pair of parallel red lines in Figure 4a. The interplanar crystal spacings of (111), 0.23 nm, and (200), 0.19 nm, were also measured from the FFT. Based on these values and the fundamental rules of FCC, the lattice constant of the crystal structure of hollow Ag–Pd nanoboxes is deduced to be 0.40 nm, which is between the values of the Pd crystal (0.39 nm and Ag crystal (0.41 nm).

In order to clarify the growth of spiky corners in the nanoboxes, a magnified high-resolution TEM image of the corner marked by a red square in Figure 5a and its FFT are shown in Figure 5c and d, respectively. The (111) lattice planes, marked by a pair of parallel red lines in Figure 5d, can clearly be observed, which illustrates that the spiky corner grew along the (111) lattice planes, the close-packed planes of the FCC structure. The growth along close-packed planes can achieve a stable structure with the lowest surface energy.^31,32^ This result also explains why there are (111) spots with strong intensities in Figure 5b.

A similar analysis was performed on the hollow Ag–Pd nanodendrites. Figure 5e and f display a high-resolution TEM image of a single nanodendrite and its corresponding FFT, respectively. Compared to the nanobox, the FFT in Figure 5f presents a more typical characteristic of polycrystals, forming clear rings. It illustrates that the Ag–Pd nanodendrite consists of a large number of small dendrites, which can be observed easily from the high-resolution TEM image as well. Moreover, the interplanar spacings of (111) and (200) lattices were calculated from the FFT image as well, where *d*_(111)_ = 0.2333 nm and *d*_(200)_ = 0.2027 nm. Furthermore, the lattice constant of the FCC structure of hollow Ag–Pd nanodendrites is deduced to be 0.4047 nm, which is in between the lattice constants of Pd and Ag crystals as well.

A magnified TEM image of a tiny dendrite from the region marked by a red square in Figure 5e is presented in Figure 5h to study its growth. Both the TEM image and its corresponding FFT in Figure 5g and h, respectively, verify that the small dendrite also grew along the close-packed planes of the FCC structure and (111) lattice planes.

The reason why different shapes of hollow Ag–Pd alloy nanoparticles are formed by using H_2_PdCl_4_ and Pd(acac)_2_ as precursors, respectively, may be explained by their solubility in water. Based on the previously proposed model,^23^ the surfactant mixture of CTAC and NaOL forms mixed micelles through their opposite charges on the surface of the Ag seeds. PdCl_4_^2–^ ions interact with positively charged cetyltrimethylammonium cation (CTA^+^) but repulsed by the negatively charged NaOL molecules.^33^ Based on the model, the Pd precursor is attracted by the positively charged CTAB molecules in the mixed micelles located on the solid surface region and reduced there, leading to the formation of small dendrites.

However, Pd(acac)_2_ is insoluble in water. This is why methanol was introduced to assist in its dissolution. Pd(acac)_2_ has a much weaker interaction with the charges of the surfactants due to its poor solubility in aqueous solution. Additionally, the limited solubility of Pd(acac)_2_ also restricts the reduction rate of Pd(II) ions, which extends the growth period of the Ag–Pd nanoboxes.

Additionally, the effect of the seed content on the growth of hollow Ag–Pd nanodendrites was investigated and the results are shown in Figure S3 of the ESI. It illustrates that the size of the nanodendrites decreases as the content of Ag nanocubes seed suspension increases, but the shape of the nanodendrites does not experience noticeable change. Besides H_2_PdCl_4_ and Pd(acac)_2_, palladium(II) acetate (Pd(acetate)_2_) and sodium tetrachloropalladate (Na_2_PdCl_4_) were also used as Pd precursors to grow hollow Ag–Pd nanoparticles. The TEM images in Figure S5 of the ESI verify that these two Pd precursors result in the growth of nanodendrites as well. This result infers that the solubility of palladium salt precursors is a crucial parameter in achieving different Ag–Pd particle morphologies. Finally, in order to gain insights into the effect of CTAC in the growth of Ag–Pd nanostructures, we replaced CTAC with potassium halide salts (i.e., KF, KCl, KBr, and KI) under the same stochiometric ratio while H_2_PdCl_4_ was used as a precursor. In the presence of a mixture of NaOL and halide ions, substantial shape degradation of the nanocube seed morphology was observed with concomitant formation of small nanoparticles, as shown in Figure S6 of the ESI. This result is supported by our previous efforts identifying the shortcomings of the colloidal stability of nanoparticles in the presence of CTAC/NaOL binary solution, which revealed that the stoichiometry of cationic and anionic surfactants is crucial for nanoparticle morphology control.^23,34^

## Conclusions

In this work, hollow Ag–Pd alloy nanodendrites and nanoboxes were synthesized successfully by using H_2_PdCl_4_ and Pd(acac)_2_, respectively, as precursors. It illustrates that the type of precursors influences the shape of final nanoparticles. These hollow Ag–Pd nanoparticles were further studied by TEM techniques. The experimental results confirm that the Ag element remains in the Pd lattice of the hollow Ag–Pd nanoparticles during the growth process, forming alloys. There is 57% of Ag in the nanodendrites and 59% of Ag in the nanoboxes by atomic percent. These Ag–Pd alloys still form FCC lattice structures with the lattice constants between the constants of Ag and Pd crystals, which is 0.4047 nm for the nanodendrite and 0.3991 nm for the nanobox. Additionally, the high-resolution TEM results verify that the small dendrites of the nanodendrites and the spiky corners of the nanoboxes both grew along the close-packed (111) lattice planes to achieve a stable structure with the lowest surface energy.

## Supporting Information Available

TEM images of hollow Ag–Pd nanodendrites and nanoboxes at different growth times; histograms of the size distribution of Ag nanocubes, hollow Ag–Pd nanodendrites, and nanoboxes; atomic percent of Ag and Pd in hollow Ag–Pd nanodendrites and nanoboxes detected by energy-dispersive X-ray spectroscopy from four regions and their average percent; extinction spectra and TEM images of hollow Ag–Pd nanodendrites synthesized by adding 5, 25, and 100 μL of Ag seed suspension, respectively; histograms of the size distribution of the hollow Ag–Pd nanodendrites; TEM images of hollow Ag–Pd nanodendrites synthesized by using palladium(II) acetate and sodium tetrachloropalladate as Pd precursors; TEM images of Ag–Pd particles prepared using the standard procedure except that no CTAC was replaced by the same molar concentration of KF, KCl, KBr, and KI (PDF)
