# Supplementary material for: The Effect of the Pd Precursors on the Shape of Hollow Ag–Pd Alloy Nanoparticles Using Ag Nanocubes as Seeds
Source: Langmuir. 2023 Jul 28;39(32):11268–73. doi: 10.1021/acs.langmuir.3c00799 (PMC10433512; doi:10.1021/acs.langmuir.3c00799)
Supplement: Supplementary file 1 — la3c00799_si_001.pdf [file la3c00799_si_001.pdf]

Supporting Information for

The Effect of the Pd Precursors on the Shape of Hollow Ag-Pd Alloy Nanoparticles Using Ag Nanocubes as Seeds

Xin Wen [a‡], Seyed Amirabbas Nazemi [b,c‡], Robson Rosa da Silva *[a,d],

Kasper Moth-Poulsen *[a,e,f,g]

a. Department of Chemistry and Chemical Engineering, Chalmers University of Technology, SE-412-96 Gothenburg, Sweden.
b. Department of Physics, Engineering, Earth, Environmental sciences, and Mechanics, University of Grenoble Alpes, 38400 Saint Martin d'Hères, France.
c. School of Life Science, University of Applied Sciences and Arts Northwestern Switzerland, Hofackerstrasee 30, Muttenz CH-4132, Switzerland.
d. NanoScientifica Scandinavia AB, Stena Center, Studio 4166, 41 292 Gothenburg, Sweden
e. The Institute of Materials Science of Barcelona, ICMAB-CSIC, Bellaterra, 08193 Barcelona, Spain.
f. Catalan Institution for Research & Advanced Studies, ICREA, Pg. Llu´ıs Companys 23, 08010 Barcelona, Spain.
g. Department of Chemical Engineering, Universitat Politècnica de Catalunya, EEBE, Eduard Maristany 10–14, 08019 Barcelona, Spain

*E-mail: kasper.moth-poulsen@chalmers.se

*E-mail: robson.silva@nanoscientifica.com

List of Contents

Figure S1. TEM images of hollow Ag-Pd nanodendrites and nanoboxes at different growth time.

Figure S2. Histograms of the size distribution of Ag nanocubes, hollow Ag-Pd nanodendrites and nanoboxes.

Table S1. Atomic percent of Ag and Pd in hollow Ag-Pd nanodendrites and nanoboxes detected by energy dispersive X-ray spectroscopy from four regions and their average percent.

Figure S3. Extinction spectra and TEM images of hollow Ag-Pd nanodendrites synthesized by adding 5, 25 and 100 µL of Ag seed suspension, respectively.

Figure S4. Histograms of the size distribution of the hollow Ag-Pd nanodendrites displayed in Figure S3.

Figure S5. TEM images of hollow Ag-Pd nanodendrites synthesized by using palladium(II) acetate and sodium tetrachloropalladate as Pd precursors.

Figure S6. Figure S6. TEM images of Ag-Pd particles prepared using the standard procedure except that CTAC was replaced by the same molar concentration of (A) KF, (B) KCl, (C) KBr, and (D) KI.

Figure S1 shows the TEM images of hollow Ag-Pd nanodendrites and nanoboxes at different growth time. The nanodendrites growing for 30 minutes in Figure S1a have a similar size and shape as the nanodendrites growing for 4 hours in Figure S1b, indicating that this growth process went very quickly. However, comparing Figures S1c and d, apparent growth of these nanoboxes can be observed from 4 hours to 22 hours. It illustrates that the Ag-Pd nanoboxes need longer time than the nanodendrites to complete the growth.

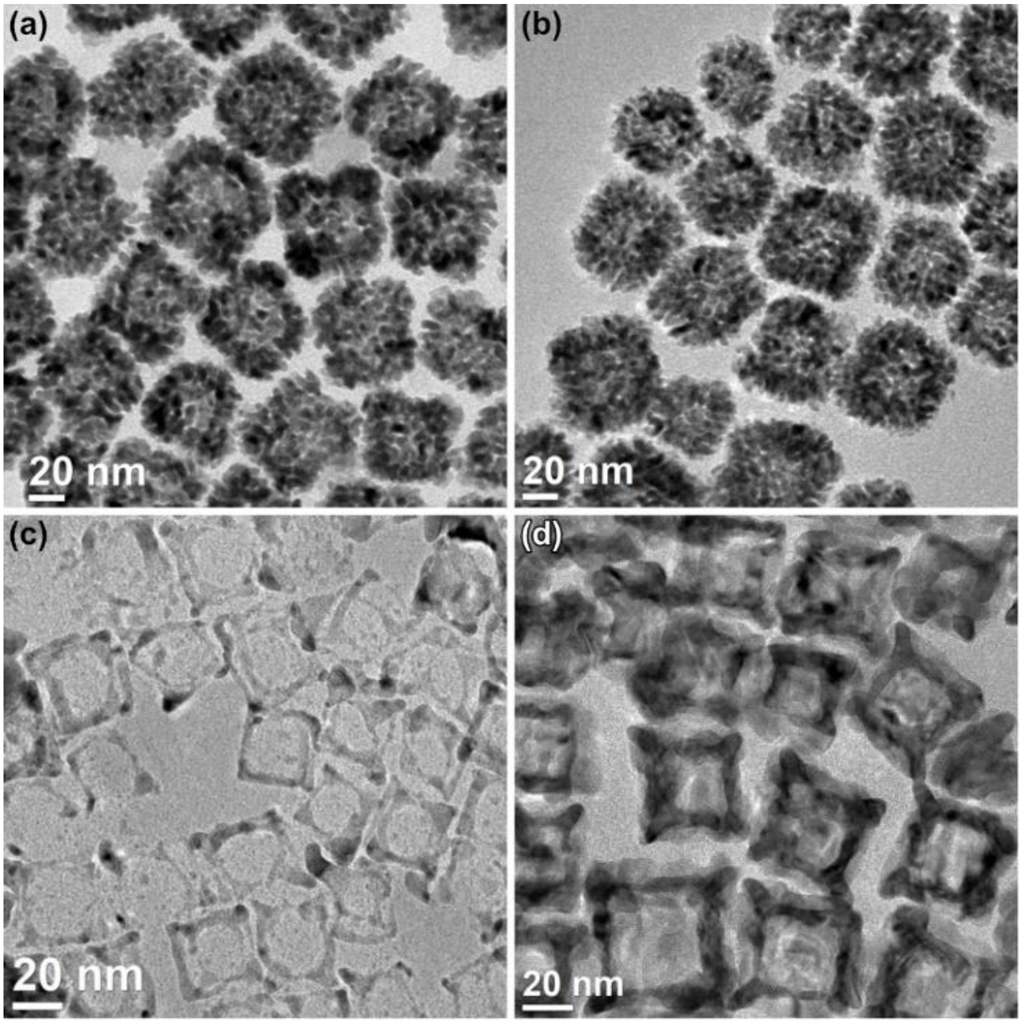

Figure S1. Hollow Ag-Pd nanoparticles at different growth time. (a) Nanodendrites, 30 minutes. (b) Nanodendrites, 4 hours. (c) Nanoboxes, 4 hours. (d) Nanoboxes, 22 hours.

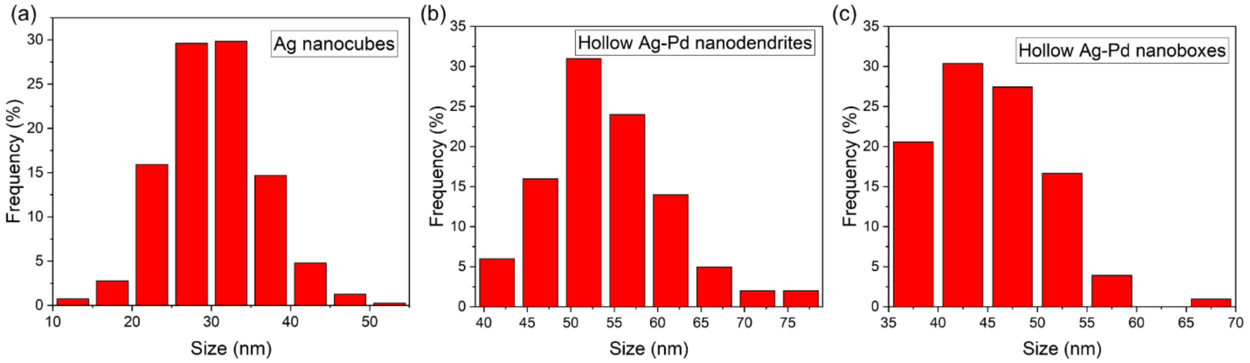

Figure S2. Histograms of the size distribution of (a) Ag nanocubes, (b) hollow Ag-Pd nanodendrites and (c) hollow Ag-Pd nanoboxes, which are corresponding to the average sizes shown in Figure 2 of the manuscript.

Table S1. Atomic percent of Ag and Pd in hollow Ag-Pd nanodendrites and nanoboxes detected by energy dispersive X-ray spectroscopy from four regions and their average percent.

Atomic percent	Region 1	Region 2	Region 3	Region 4	Average
Hollow Pd nanodendrites					
Pd	42%	40%	39%	52%	43%
Ag	58%	60%	61%	48%	57%
Hollow Pd nanoboxes					
Pd	34%	28%	56%	47%	41%
Ag	66%	72%	44%	53%	59%

In Figure S3, hollow Ag-Pd nanodendrites were synthesized by adding 5, 25 and 100 μL of Ag seed suspension in order to study the effects of the volume of seed suspension on the shape and size of Ag-Pd nanodendrites. Figure S3a displays the extinction spectra of three nanodendrite suspension. The extinction peak has a slight red shift from the wavelength of 345 nm to 351 nm as the volume of Ag seed suspension is increasing, indicating that the size of Ag-Pd nanodendrites is changing slightly. The average sizes of the nanodendrites are 73.5 ± 7.4 nm (5 μL of Ag seed suspension), 59.5 ± 7.8 nm (25 μL of Ag seed suspension) and 56.2 ± 6.5 nm (100 μL of Ag seed suspension). As the volume of Ag seed suspension is increasing, the size of the nanodendrites is decreasing slightly. However, the volume of the seed suspension does not have obvious effect on the shape of hollow Ag-Pd nanodendrites.

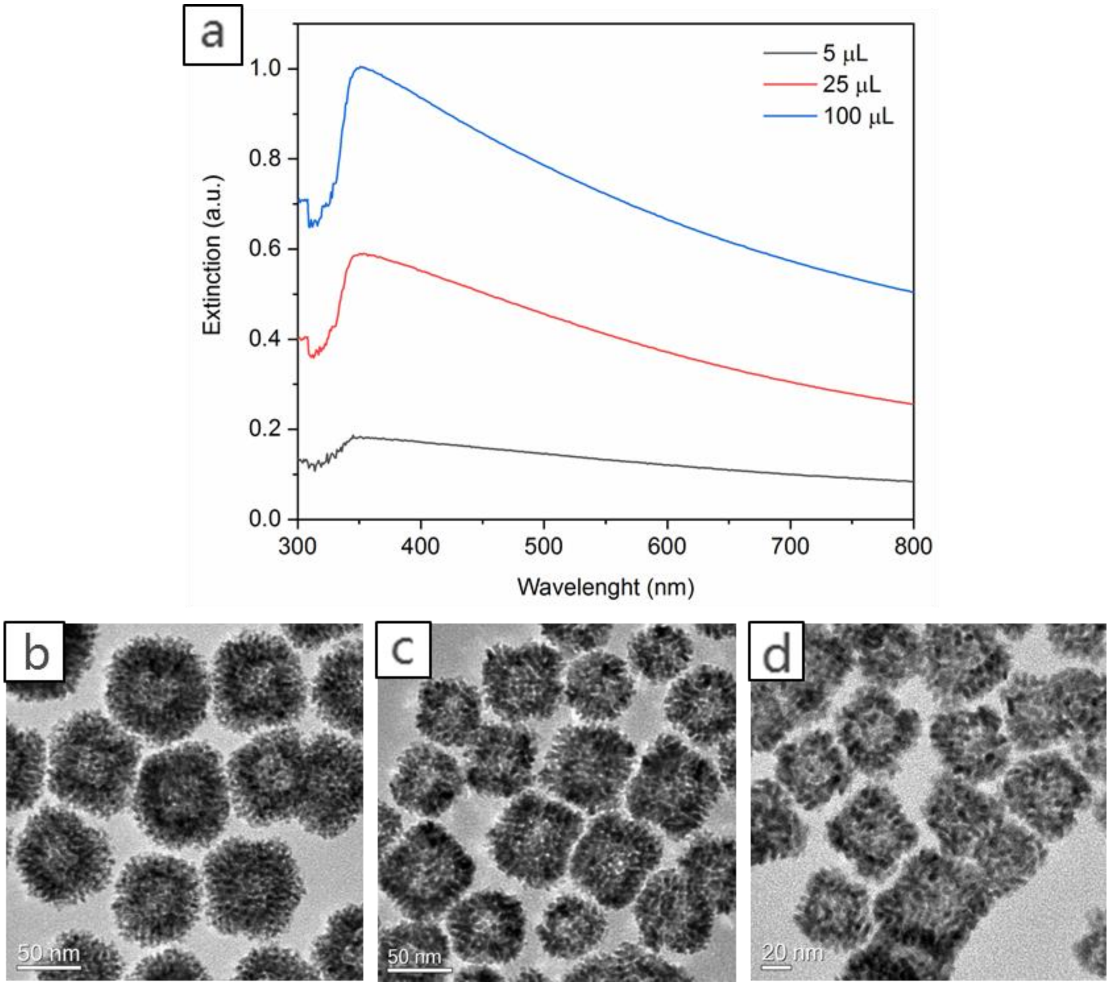

Figure S3. (a) Extinction spectra of hollow Ag-Pd nanodendrites synthesized with 5, 25 and 100 μL of Ag-nanocube seed suspension, respectively. TEM images of Pd nanodendrites using (b) 5 μL, (c) 25 μL, and (d) 100 μL of Ag seed suspension.

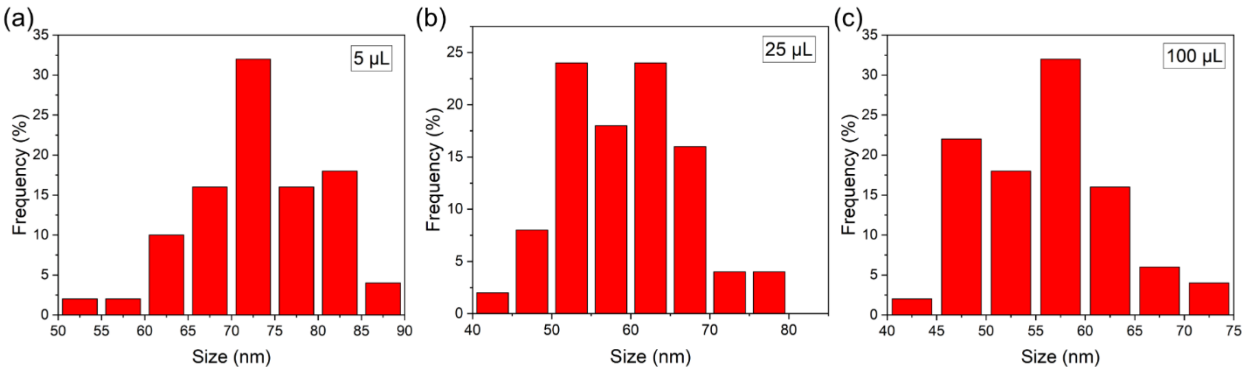

Figure S4. Histograms of the size distribution of the hollow Ag-Pd nanodendrites synthesized by adding different amount of the Ag seed suspension, corresponding to the nanoparticles shown in Figure S3. (a) 5 μL, (b) 25 μL and (c) 100 μL.

Besides H_2PdCl_4 and $Pd(acac)_2$, $Pd(acetate)_2$ and Na_2PdCl_4 tetrachloropalladate were also used as Pd precursors in the growth suspension. The TEM images in Figure S5 indicate that these two Pd precursors lead to a formation of hollow Ag-Pd nanodendrites as well.

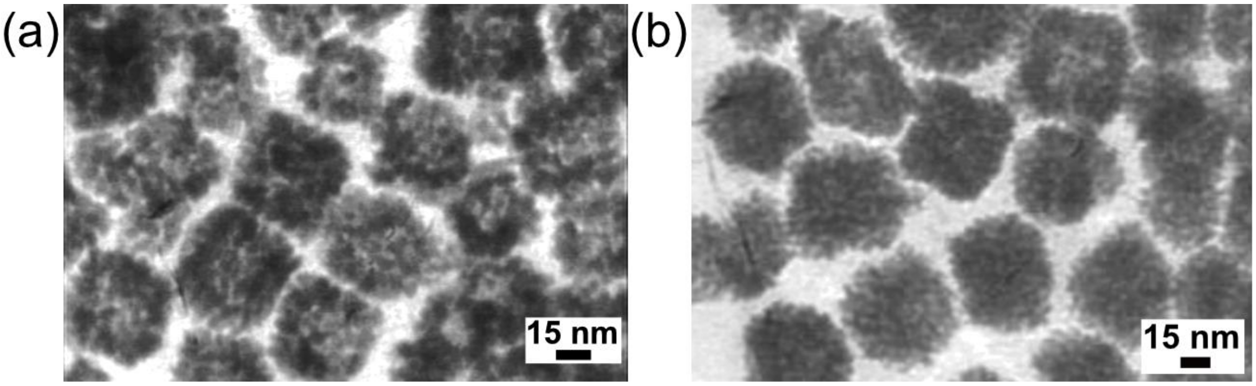

Figure S5. Hollow Ag-Pd nanodendrites synthesized by using (a) palladium (II) acetate and (b) sodium tetrachloropalladate as Pd precursors.

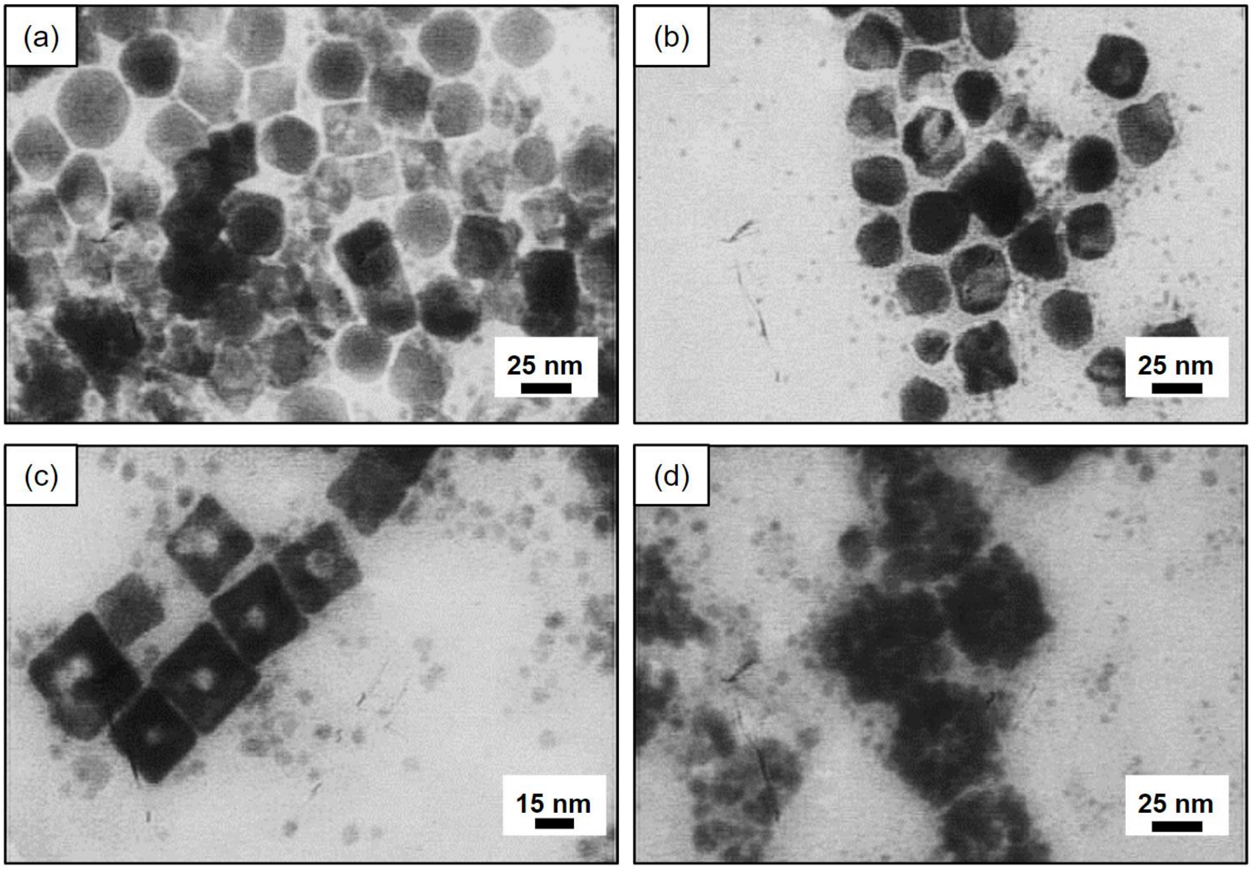

Figure S6. TEM images of Ag-Pd particles prepared using the standard procedure except that no CTAC was replaced by same molar concentration of (A) KF, (B) KCl, (C) KBr, and (D) KI.
